# Pharmacokinetic investigation of dose proportionality with a 24-hour controlled-release formulation of hydromorphone

**DOI:** 10.1186/1472-6904-7-3

**Published:** 2007-02-02

**Authors:** Gayatri Sathyan, Emily Xu, John Thipphawong, Suneel K Gupta

**Affiliations:** 1 ALZA Corporation, Mountain View, CA, USA

## Abstract

**Background:**

The purpose of this study was investigate the dose proportionality of a novel, once-daily, controlled-release formulation of hydromorphone that utilizes the OROS^® ^Push-Pull™ osmotic pump technology.

**Methods:**

In an open-label, four-way, crossover study, 32 healthy volunteers were randomized to receive a single dose of OROS^® ^hydromorphone 8, 16, 32, and 64 mg, with a 7-day washout period between treatments. Opioid antagonism was provided by three or four doses of naltrexone 50 mg, given at 12-hour intervals pre- and post-OROS^® ^hydromorphone dosing. Plasma samples for pharmacokinetic analysis were collected pre-dose and at regular intervals up to 48 hours post-dose (72 hours for the 64-mg dose), and were assayed for hydromorphone concentration to determine peak plasma concentration (C_max_), time at which peak plasma concentration was observed (T_max_), terminal half-life (t_1/2_), and area under the concentration-time curve for zero to time t (AUC_0-t_) and zero to infinity (AUC_0–∞_). An analysis of variance (ANOVA) model on untransformed and dose-normalized data for AUC_0-t_, AUC_0–∞_, and C_max _was used to establish dose linearity and proportionality.

**Results:**

The study was completed by 31 of 32 subjects. Median T_max _(12.0–16.0 hours) and mean t_1/2 _(10.6–11.0 hours) were found to be independent of dose. Regression analyses of C_max_, AUC_0–48_, and AUC_0–∞ _by dose indicated that the relationship was linear (slope, *P *≤ 0.05) and that the intercept did not differ significantly from zero (*P *> 0.05). Similar analyses with dose-normalized parameters also indicated that the slope did not differ significantly from zero (*P *> 0.05).

**Conclusion:**

The pharmacokinetics of OROS^® ^hydromorphone are linear and dose proportional for the 8, 16, 32, and 64 mg doses.

**Trial Registration:**

Clinical Trials.gov NCT00398957

## Background

Hydromorphone hydrochloride (HCl), which is available in immediate- and extended-release formulations, is a semi-synthetic opioid agonist that has been used widely for many years in the treatment of acute and chronic pain. A number of studies have demonstrated the efficacy and tolerability of hydromorphone in comparison with morphine and other opioid analgesic agents [1]. When formulated as an immediate-release preparation, hydromorphone has an elimination half-life of approximately 2 to 3 hours [2-4]. As a consequence, doses must be administered every 4 to 6 hours to ensure continuous analgesia for the patient [5].

To improve pain relief and provide convenient dosing for patients with severe chronic cancer and non-cancer pain, a novel 24-hour controlled-release formulation of hydromorphone is currently being investigated. This formulation uses the patented OROS^® ^Push-Pull™ osmotic pump delivery system developed by ALZA Corporation (Palo Alto, CA) [6-8], and a consistent release of hydromorphone over 24 hours has been demonstrated in healthy volunteers [9]. Moreover, steady-state plasma concentrations for OROS^® ^hydromorphone (Jurnista™, Janssen Pharmaceutica, N.V., Beerse, Belgium) are achieved after 48 hours (i.e., after two doses or by the third dose) and are maintained throughout the 24-hour dosing interval [10]. An initial study also has shown that the pharmacokinetics of hydromorphone are not substantially affected when OROS^® ^hydromorphone is taken immediately after a high-fat meal [11].

Co-administration of OROS^® ^hydromorphone with naltrexone, an opioid antagonist, under fasting conditions resulted in a 39% increase in C_max_, but there was no significant change in T_max_, AUC_0-t_, or AUC_0–∞ _[11]. These results indicate that blockade of opioid effects by naltrexone is useful in comparative bioavailability studies of high-dose opioids in healthy volunteers, with the assumption that all treatments are affected similarly. The objective of the present study was to evaluate the dose proportionality and linearity of OROS^® ^hydromorphone at daily doses of 8, 16, 32, and 64 mg.

## Methods

### Subjects

Study volunteers were non-smoking, healthy male and female adults between 19 and 50 years of age. Their body weight was required to be between 61 and 100 kg and within ± 10% of the recommended weight range for height and body frame (1984 Metropolitan Height and Weight Tables). Results of the baseline screen were required to be negative for drugs of abuse (cannabinoids, opiates, cocaine, ethanol, and barbiturates). Subjects were required to have no clinically significant deviations from normal in laboratory results. All participants provided written informed consent. The study was approved by the Institutional Review Board and was carried out according to the Declaration of Helsinki and subsequent revisions.

Subjects who were intolerant of, or hypersensitive to, opioid agonists or antagonists were excluded, as were those with opioid dependency. Other exclusion criteria included gastrointestinal disorders; compromised cardiac, respiratory, renal, or hepatic function; psychiatric abnormalities; and significant hematologic, metabolic, or central nervous system disorders. Study participation did not permit any subject to take any long-term medication, enzyme-altering agents, recreational drugs, or an investigational agent within 30 days of beginning the study.

### Study design and interventions

This was an open-label, randomized, four-way crossover study designed to examine the pharmacokinetic profile of once-daily OROS^® ^hydromorphone for dose proportionality after administration of a single oral dose of 8, 16, 32, and 64 mg.

Based on the assumption that the within-subject variability is less than 20% (value guided by variability in exposure following immediate-release hydromorphone) and that there is a 5% difference between treatments, a sample size of 30 subjects was estimated to provide 80% power to demonstrate equivalence at the 0.05 level of significance.

Subjects received each of the four treatments (OROS^® ^hydromorphone 8, 16, 32, and 64 mg, given after a 10-hour overnight fast), with a 7-day washout period between treatments. The order in which treatments were received was determined according to the predetermined randomization schedule. Naltrexone 50 mg was administered 12 hours before, with, and 12 hours after OROS^® ^hydromorphone in all groups, with an additional 50-mg dose of naltrexone administered 24 hours after the 64-mg dose of OROS^® ^hydromorphone. Naltrexone was administered to minimize adverse events following the higher doses of OROS^® ^hydromorphone in these opioid-naïve subjects, and was given concomitantly with each dose level of OROS^® ^hydromorphone to facilitate dose-proportionality comparisons.

### Plasma sampling

Plasma samples for pharmacokinetic analysis were collected pre-dose (time 0) and at 2, 4, 6, 8, 10, 12, 16, 20, 24, 30, 36, 42, and 48 hours post-dose. Additional samples were taken at 56, 64 and 72 hours after the 64-mg dose. Plasma hydromorphone concentrations were measured using a validated LC/MS/MS method (CEDRA Corporation, Austin, TX) covering a range of 0.05 to 10 ng/mL. Calibration standards prepared for each of the sample sets were used to calculate the inter-day precision of the assay. The coefficients of variation for the standards ranged from 1.7% to 9.9%. The absolute deviations ranged from 0.05% to 2.6%.

Based on the measured hydromorphone concentration, the following parameters were calculated: peak plasma concentration (C_max_), time at which peak plasma concentration was observed (T_max_), terminal half-life (t_1/2_), and the area under the concentration-time curve from time 0 to time t (AUC_0-t_) and from time zero to infinity (AUC_0–∞_). The non-compartmental pharmacokinetic parameters described above were estimated using macros built in Excel (Microsoft, Redmond, WA).

### Statistical analysis

Untransformed and log-transformed (ln) data for C_max_, AUC_0-t _and AUC_0–∞ _were analyzed using an appropriate analysis of variance (ANOVA) regression model to establish dose linearity and dose proportionality. All tests were two-sided at the 0.05 level of significance. T_max _was analyzed non-parametrically, without dose-normalization, using the Wilcoxon matched-pairs test for each pairwise comparison; the 95% confidence interval (CI) for the difference in treatment medians was constructed. Data for t_1/2 _were summarized using descriptive statistics. The apparent elimination-rate constant (K) for each subject was estimated by linear regression of the log-transformed concentration during the terminal log-linear decline phase of the curve. Terminal half-life was estimated as 0.693/K.

## Results

### Subjects

Thirty-two healthy volunteers were enrolled in the study, 8 in each of four treatments, with at least 24 subjects expected to complete the study. They were primarily male (63%) and Caucasian (81%), with a mean age of 33 years (Table 1). The study was completed by 31 subjects; one subject discontinued for personal reasons, after completing the first phase of treatment (64-mg dose).

**Table 1:** Baseline characteristics

**Characteristic**	**All Participants (*n *= 32)**
Sex, *n *(%)	
Male	20 (62.5)
Female	12 (37.5)
Race, *n *(%)	
Caucasian	26 (81.3)
Asian	3 (9.4)
Black	1 (3.1)
Hispanic	1 (3.1)
American Indian	1 (3.1)
Age (years)	
Mean	33
Range	20–50
Height (cm)	
Mean	175
Range	163–191
Weight (kg)	
Mean	76.4
Range	61.4–96.4

### Pharmacokinetics

**Figure 1:**
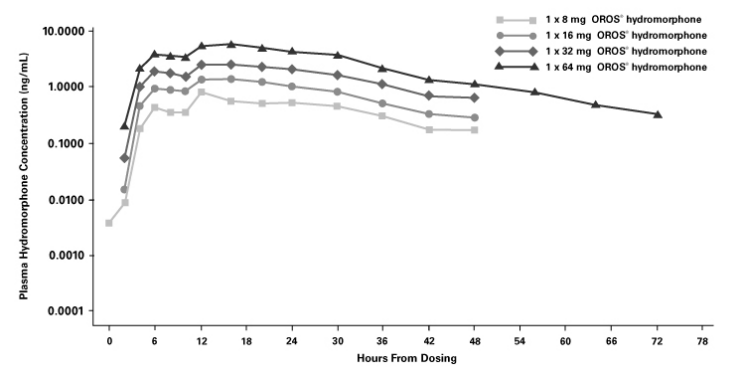
Mean plasma hydromorphone concentrations over time after administration of single-dose OROS^® ^hydromorphone.

**Figure 2:**
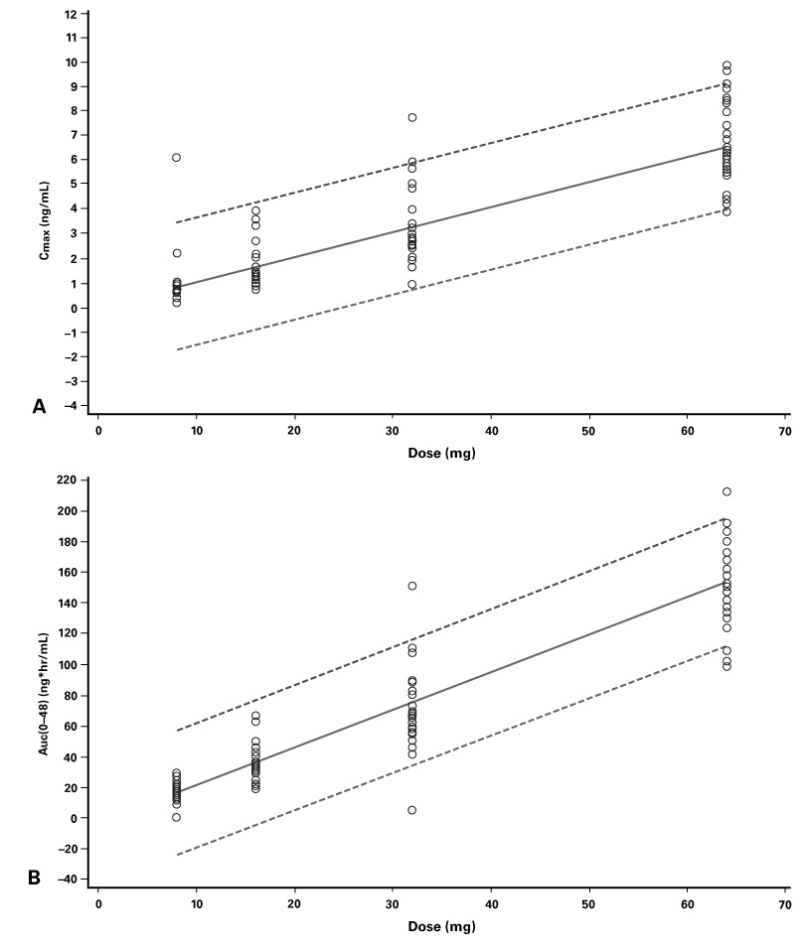
Analyses of dose linearity with OROS^® ^hydromorphone: (A) C_max_/dose; (B) AUC_0–48_/dose. Dashed lines represent 95% CI.

The plasma concentration-time profiles of the four OROS^® ^hydromorphone doses tested are shown in Figure 1. Following a single oral dose of OROS^® ^hydromorphone, plasma mean concentrations gradually increase over 6 to 8 hours, and thereafter are sustained at or near maximum levels up to approximately 30 hours post-dose. The means of untransformed pharmacokinetic parameters and the medians of T_max _are shown in Table 2. Maximum plasma hydromorphone concentrations were achieved approximately 12 to 16 hours after administration, with no significant dose effect observed. Mean values for t_1/2 _were similar for the various doses (10.6–11.0 hours). Analysis of C_max_, AUC_0-t_, and AUC_0–∞ _by dose indicated that the relationship was linear (*P *≤ 0.05) and that the intercept did not differ significantly from zero (*P *> 0.05; Figure 2).

**Table 2:** Untransformed pharmacokinetic parameters after administration of OROS^® ^hydromorphone 8, 16, 32, and 64 mg (*n *= 31)

**Parameter**	**8 mg**	**16 mg**	**32 mg**	**64 mg**
C_max _(ng/mL)				
Mean	0.929	1.69	3.25	6.61
SD	1.01	0.78	1.37	1.75
T_max _(hour)				
Median	12.0	16.0	16.0	16.0
Range	4.0–30.0	6.0–30.0	4.0–24.0	6.0–30.0
AUC_0–48 _(ng·hr/mL)				
Mean	18.1	36.5	72.2	156
SD	5.8	11.3	24.3	30.6
AUC_0–∞ _(ng·hr/mL)				
Mean	19.5	40.8	80.3	178.7
SD	5.9	13.7	29.6	35.2
t_1/2 _(hour)				
Mean	10.6	10.3	11.0	10.9
SD	4.3	2.4	3.2	3.8

**Figure 3:**
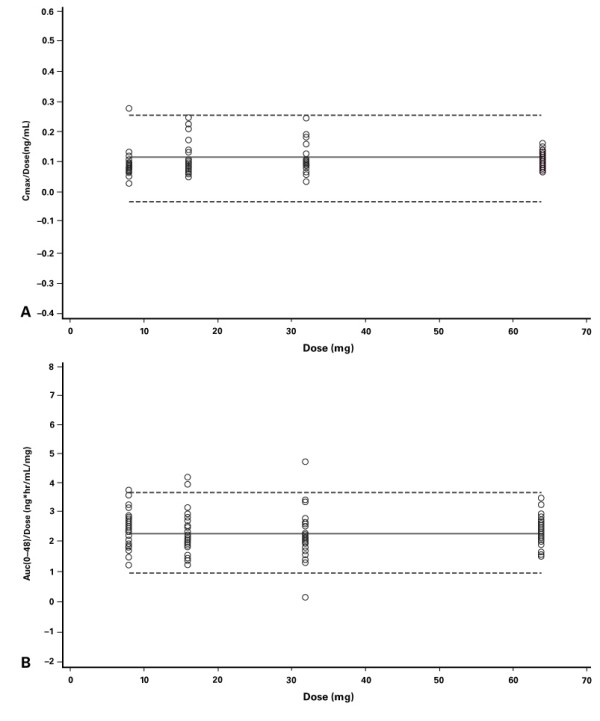
Analyses of dose proportionality with OROS^® ^hydromorphone: (A) C_max_/dose; (B) AUC_0–48_/dose. Dashed lines represent 95% CI.

Mean dose-normalized pharmacokinetic parameters for OROS^® ^hydromorphone after administration of 8, 16, 32, and 64 mg doses are shown in Table 3. C_max _and AUC increased linearly and in a manner proportional to the dose of OROS^® ^hydromorphone. The slopes of dose-normalized C_max _and AUC vs. dose did not differ significantly from zero (*P *> 0.05; Figure 3). Inter-subject variability in pharmacokinetic parameters was similar across the doses except for high variability of C_max _following the 8-mg dose. This was mainly due to one subject with a high concentration (>5 times the mean). When this subject was excluded, C_max _variability for the 8-mg dose was similar to the other doses. No significant gender-by-treatment interactions were observed (ANOVA model; data not shown).

**Table 3:** Mean (SD) of the dose-normalized pharmacokinetic parameters after administration of OROS^® ^hydromorphone 8, 16, 32, and 64 mg (*n *= 31)

**Parameter**	**8 mg**	**16 mg**	**32 mg**	**64 mg**
C_max_, ng/mL/mg	0.116 (0.127)	0.106 (0.049)	0.102 (0.043)	0.103 (0.027)
AUC_0–48_, ng·hr/mL/mg	2.26 (0.72)	2.28 (0.71)	2.26 (0.76)	2.44 (0.48)
AUC_0–∞_, ng·hr/mL/mg	2.44 (0.74)	2.55 (0.86)	2.51 (0.93)	2.79 (0.55)

### Safety

At least one adverse event was experienced by 21 of the 32 subjects (66%). All events were of mild or moderate intensity, and none were considered serious. Headache, asthenia, and nausea were the most common adverse events, occurring in 31%, 28%, and 28% of patients, respectively, during one or more of the treatment periods. The adverse events for each dose group are shown in Table 4. No treatment-related trends were noted with regard to vital signs, electrocardiogram results, or clinical laboratory data.

**Table 4:** Adverse events occurring in ≥10% of subjects in any treatment group

**Event, *n *(%)**	**8 mg (*n *= 31)**	**16 mg (*n *= 31)**	**32 mg (*n *= 31)**	**64 mg (*n *= 32)**
Asthenia	2 (6)	5 (16)	3 (10)	5 (16)
Nausea	4 (13)	5 (16)	3 (10)	3 (9)
Headache	2 (6)	3 (10)	5 (16)	3 (9)
Chest pain	0 (0)	0 (0)	3 (10)	2 (6)

## Discussion

The results of this study indicate that plasma hydromorphone concentrations and overall exposure to hydromorphone are proportional to the administered dose (over the 8- to 64-mg dose range) with OROS^® ^hydromorphone. The time to achieve maximum plasma concentration was independent of dose. Near-maximum plasma concentrations were reached approximately 6 hours after dosing, and plasma concentrations were maintained at or near maximum levels throughout a 30-hour period, consistent with once-daily dosing. Beginning 24 to 30 hours post-dose, plasma hydromorphone concentrations declined slowly, with an apparent terminal half-life of approximately 10 hours. This is longer than the half-life of immediate-release hydromorphone (2–3 hours), which has been determined from studies with intravenous formulations [2-4]. The present study included plasma sampling for up to 72 hours post-dose, and it was designed to characterize both the controlled-release and the post-absorptive elimination phases of the drug. The apparent terminal half-life observed in this study is similar to that seen in a study designed to assess the effects of food intake on the pharmacokinetics of OROS^® ^hydromorphone [11]. The observed plasma profile with concentration maintained over 24 hours supports the proposed once-daily administration of OROS^® ^hydromorphone.

An exploratory analysis suggested no influence of gender on the pharmacokinetics of OROS^® ^hydromorphone for the dose range studied. Although limited, these data do suggest that there are no clinically relevant differences between males and females with respect to the pharmacokinetics of OROS^® ^hydromorphone.

Safety results were consistent for all four OROS^® ^hydromorphone doses, indicating no dose relationship with the incidence of adverse events. Adverse events were consistent with those expected for an opioid agonist and antagonist and primarily affected the digestive and central nervous systems. No serious adverse events were reported during the study.

## Conclusion

Plasma concentrations of OROS^® ^hydromorphone and its pharmacokinetic parameters were found to be proportional to the orally administered dose over the dose range studied (8 mg to 64 mg). Plasma concentrations achieved the maximal level by approximately 16 hours after single administration, independently of dose, and remained near that level for up to 30 hours. Adverse events were consistent with those expected for an opioid agonist and antagonist.

## Competing interests

The authors declare that they have no competing interests.

Financial Competing Interests

In the past five years none of the authors of this manuscript have received reimbursements, fees, funding, or salary from an organization that may in any way gain or lose financially from publication of this manuscript, either now or in the future.

The authors of this manuscript do hold stock in ALZA Corporation but the stock price is not affected by the product alone or the publication.

There are no patents filed based on the data disclosed in this publication. The authors have not received reimbursements, fees, funding, or salary from an organization that holds or has applied for patents relating to the content of the manuscript.

The authors do not have any other financial competing interests.

The authors do not have any non-financial competing interests (political, personal, religious, academic, ideological, intellectual, commercial or any other) to declare in relation to this manuscript.

## Authors' contributions

GS reviewed the pharmacokinetic and statistical results and authored the related sections. EX performed all the pharmacokinetic and statistical analysis. SKG participated in the conception of OROS hydromorphone product and contributed to the design of the study. JT reviewed the safety results and authored the related sections. All authors read and approved the final manuscript. All authors wish to acknowledge Philip Sjostedt and PharmaGenesis, Inc., in preparation of this manuscript.

## Pre-publication history

The pre-publication history for this paper can be accessed here:


